# Controlling GaN-Based Laser Diode Performance by Variation of the Al Content of an Inserted AlGaN Electron Blocking Layer

**DOI:** 10.3390/nano14050449

**Published:** 2024-02-29

**Authors:** Yuhui Chen, Daiyi Jiang, Chunmiao Zeng, Chuanxiong Xu, Haoran Sun, Yufei Hou, Mei Zhou

**Affiliations:** 1 Department of Applied Physics, China Agricultural University, Beijing 100083, China; yuhuichen@cau.edu.cn (Y.C.); xcxxx@cau.edu.cn (C.X.); sunhaoran047@163.com (H.S.); 2 College of Science, China Agricultural University, Beijing 100083, China; 2022310020110@cau.edu.cn (D.J.); 2022310020121@cau.edu.cn (C.Z.); 3 State Key Laboratory on Integrated Optoelectronics, Institute of Semiconductors, Chinese Academy of Sciences, Beijing 100083, China; houyufei@semi.ac.cn

**Keywords:** GaN-based LD, Al content, electron blocking layer, electron leakage

## Abstract

The leakage of the electronic current of a laser diode (LD) has some significant influences on the performance of the LD. In this study, commercial simulation software LASTIP is used to numerically evaluate the performances of LDs by using different wavelengths and Al contents of the electron blocking layer (EBL). These LDs a adopt multilayer structure, which contains cladding layers, waveguide layers, multiple quantum well layers, contact layers and an Al_x_Ga_1−x_N EBL. The influence mechanism of EBL is theoretically examined by analyzing the simulated performances. It is found that for short-wavelength violet LDs, the electrical and optical properties of the LD will reach the optimum state when the Al content (x) in the EBL is nearly 0.25. For long-wavelength green LDs, it will achieve optimum electrical and optical properties when the Al content in the EBL is as low as possible. We also compare the simulation results of LDs with emission wavelengths in the range of violet and green, including blue cyan, for a more general evaluation. According to the simulated results, it is verified that the influence of the EBL’s Al content on LD performance enhances as the wavelength increases.

## 1. Introduction

The band gap of GaN-based nitrides is continuously adjustable in the range of 0.7 eV to 6.2 eV [1,2], and this excellent physical property allows GaN-based LDs to have broad application prospects in many fields, such as high-density optical storage, full-color display, chemical sensors and portable projectors [3,4,5,6]. In addition, III–V nitride nanostructures have excellent electrical, energy band and structural properties. In 2023, Gianfranco Sfuncia et al. investigated the properties of the III–V nitride nanostructures through first-principles calculations based on density functional theory, which contributes to the construction of more stable III–V multilayer nanostructures [7]. However, due to the high degree of dislocation usually produced by nitride thin films, the performance of the device deteriorates. Manoel Alved Machado Filho et al. obtained electronic properties of the structure of several III–V nitrides by modeling based on density functional theory (DFT), which contributes to the achievement of on-demand growth of nanorods [8]. But at present, GaN-based LDs still face the problem of electron leakage, which can lead to a decrease in the luminous efficiency of LDs. The heat generated by the overflow current increases the junction temperature of LDs and reduce their operating lifetime [9]. In order to reduce electron current leakage, Nakamura et al. first proposed the method of inserting an electron blocking layer (EBL) after the growth of quantum well in LDs in 1996 [10], and this method has been widely used to suppress electron leakage since then. In 2010, Yen-Kuang Kuo et al. compared the role of EBL in iii-nitride blue LDs and LEDs. Compared with LEDs, since GAN-based LDs have a larger injection current, carriers in the active region are more likely to overflow outside the active region, especially electrons. In this case, the EBL becomes more crucial for achieving improved device performance, and researchers found that for blue LDs, the higher the Al content (up to 20%), the better the performance [11]. For LDs with shorter wavelengths, like blue–violet light, the weak confinement ability of electrons in the shallower quantum wells can cause serious electron current overflow from the active region of the LD. On the other hand, since the polarization electric field between the quantum barrier layer (QB) and the EBL can induce the downward bending of the energy band, the accumulation of electrons is aggravated and the height of the EBL’s effective potential barrier is reduced, which can lead to serious electron current leakage [12,13]. For longer-wavelength LDs, such as green LDs, although they have deeper quantum wells, the likelihood of carrier leakage is also intensified with an increase in current injection. This problem is mainly related to the low injection efficiency of hole current, which is influenced by the EBL [14]. Generally, compared with electrons, holes have a larger effective mass and lower mobility; therefore, they are not very efficient to inject from the EBL into quantum wells. Meanwhile, low hole injection also increases the leakage of electrons [15,16]. In order to reduce electron leakage, people researchers have explored the optimization of the electron barrier. In these studies, researchers confirmed that the Al content in Al_x_Ga_1−x_N EBL can be optimized for suppression of carrier leakage in the quantum well structure [17,18,19]. However, there are few studies on the regulating mechanism of Al content of the AlGaN EBL in various wavelength ranges and its effects on LD performance, resulting in a lack of horizontal comparisons and analyses.

In this study, in order to horizontally compare the influence of the Al content of the EBL on LDs with different wavelengths, short-wavelength violet (λ = 409 nm) and long-wavelength green (λ = 540 nm) LDs are selected as examples. Their performances are first evaluated and analyzed because they are significantly different in the emission wavelength. In addition, the performance of blue (λ = 446 nm) and cyan (λ = 485 nm) LDs, which have wavelengths between the above-mentioned violet and green LDs, are also be simulated and evaluated to verify the influence of the wavelength-dependent property. By using the same device structure for violet, blue, cyan and green LDs, the Al content of the LDs’ Al_x_Ga_1−x_N EBL is adjusted to vary within a gradient step from 0.03 to 0.35, and the percentage of electron leakage current (PELC), electrical properties and optical properties of LDs are analyzed through a systematical study of the regulating mechanism of laser diode performance exerted by the Al content of Al_x_Ga_1−x_N EBL. The research results have certain guiding significance for the design of practical laser diodes to suppress electron leakage and improve device performance.

## 2. Laser Structure and Simulation Parameters

**Figure 1:**
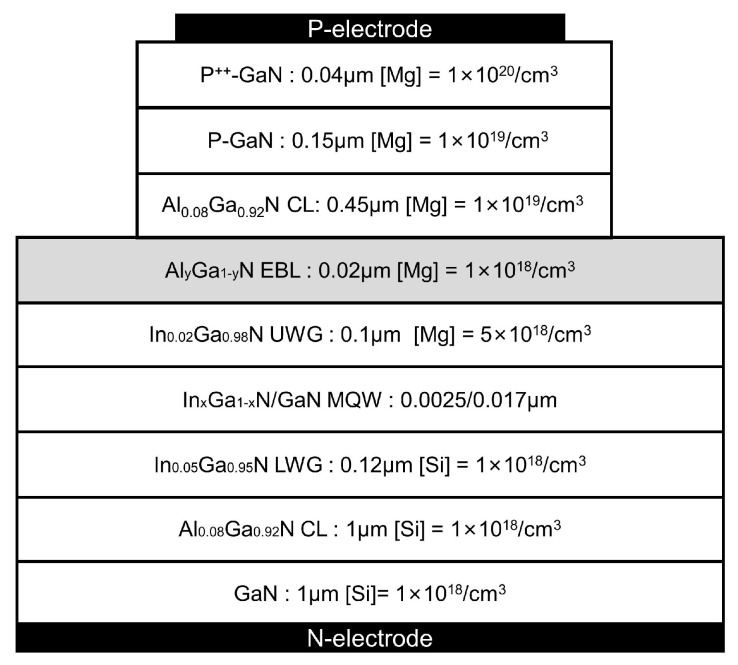
Schematic diagram of the GaN-based LD structure. [Si]: Si doping level. [Mg]: Mg doping level. CL: cladding layer. LWG: lower waveguide layer. UWG: upper waveguide layer. EBL: electron blocking layer.

The details of the basic structure parameters of a GaN-based LD, such the layer thicknesses and doping level of each layer, are shown in Figure 1. The ridge width and cavity length of the LD devices are 3 μm and 600 μm, respectively. The LD structure consists of a 1 μm GaN substrate (Si: 1 × 10^18^/cm^3^), a 1 μm Al_0.08_Ga_0.92_N cladding layer (Si: 1 × 10^18^/cm^3^), and a 0.12 μm In_0.05_Ga_0.95_N lower waveguide layer (Si: 1 × 10^18^/cm^3^), a periodic multiple quantum well (MQW) active region, a 0.1 μm In_0.02_Ga_0.98_N upper waveguide layer (Mg: 5 × 10^18^/cm^3^), a 0.02 μm Al_y_Ga_1−y_N electron blocking layer (Mg: 1 × 10^18^/cm^3^), a 0.45 μm Al_0.08_Ga_0.92_N cladding layer (Mg: 1 × 10^19^/cm^3^), a 0.15 μm P-GaN layer (Mg: 1 × 10^19^/cm^3^) and a 0.04 μm heavily doped p^++^-GaN contact layer (Mg: 1 × 10^20^/cm^3^). The structure of LDs with different wavelengths is the same for all layers, except the In content of In_x_Ga_1−x_N quantum well layers and the Al content of the Al_x_Ga_1−x_N EBL. The In content of the In_x_Ga_1−x_N QW layers of violet, blue, cyan and green LDs is set to be 0.19, 0.22, 0.29 and 0.37, respectively. Additionally, the Al content of the Al_y_Ga_1−y_N of EBL varies from 0.03 to 0.35.

This study uses Crosslight’s LASTIP software to simulate the output performance of the above-mentioned GaN-based LDs with four different wavelengths. This software is a powerful 2D semiconductor laser simulation software that can solve the Poisson equation, the Schrodinger equation and the current continuity equation by the finite element method to obtain the photoelectric characteristics of LDs [20,21]. During the calculation, both P-type and N-type electrodes are set as ideal ohmic contact, and only 25% of the theoretical value of the polarization field is applied [22]. The LD operating temperature is set to be 300 K. The absorption coefficient of different layers is set as a function of doping concentration, and the function expression is as follows [23]:

(1) $$ncentration\left({\mathrm{cm}}^{-3}\right)/\left(110^{19}\left({\mathrm{cm}}^{-3}\right)\times 50\left({\mathrm{cm}}^{-1}\right)\right)$$

Moreover, the refractive indices of InGaN-based and AlGaN-based alloys are calculated by an approximate method, and their expressions are as follows [16]:

(2) $$n\left(In_{x}Ga_{1-x}N\right)=\left[n\left(InN\right)-n\left(GaN\right)\right]\cdot x+n\left(GaN\right)$$

(3) $$n\left(Al_{x}Ga_{1-x}N\right)=\left[n\left(AlN\right)-n\left(GaN\right)\right]\cdot x+n\left(GaN\right)$$

It is noted that the *n* values are wavelength-dependent. For the violet LD (λ = 409 nm), the refractive indices of InN, GaN and AlN are 3.7918, 2.4850 and 1.9283, respectively. For the blue LD (λ = 446 nm), the refractive indices of InN, GaN and AlN are 2.8848, 2.4581 and 1.9006, respectively. For the cyan LD (λ = 485 nm), the refractive indices of InN, GaN and AlN are 2.5075, 2.4541 and 2.1706, respectively. For the green LD (λ = 540 nm), the refractive indices of InN, GaN and AlN are 2.8796, 2.435 and 2.2475, respectively.

## 3. Results and Discussion

### 3.1. Violet and Green LDs

#### 3.1.1. Electron Leakage Current of Violet and Green LDs

**Figure 2:**
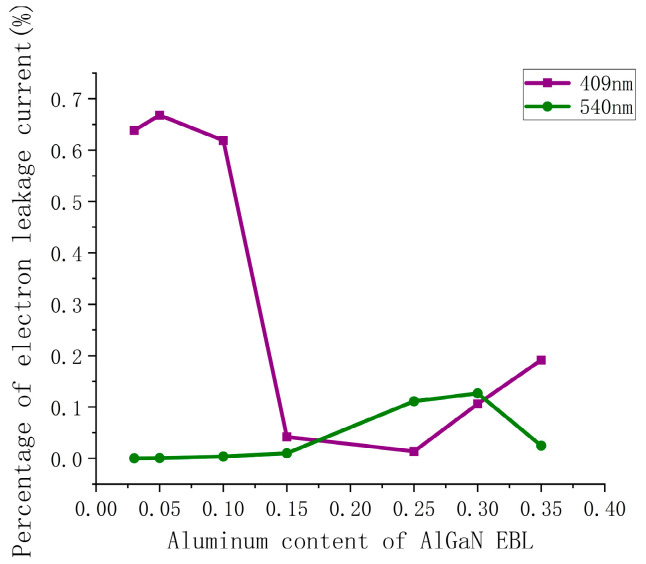
Relationship between the percentage of electron current leakage (PELC) of violet and green LDs versus the Al content of the AlGaN EBL.

First, the effect of Al_x_Ga_1−x_N EBL on the percentage of electron leakage current (PELC) of violet and green LDs is simulated when the Al content (x) is changed in a range between 3% and 35%. As shown in Figure 2, for the violet LD, the PELC decreases from 63.9% to 1.4% as a whole when the Al content of AlGaN EBL increases from 3% to 25%. When the Al content of AlGaN EBL is greater than 0.25, the PELC increases from 1.4% to 19.2%. On the other hand, for the green LD, as the Al content of AlGaN EBL increases, the PELC increases from 0.03% to 12.65% at first, but when the Al content of AlGaN EBL exceeds 0.3, the PELC begins to drop from 12.65% to 2.46%. The results show that as the Al content of AlGaN EBL increases, the variation trend of violet and green LD electron current leakage is different, which means that the Al content of AlGaN EBL has a completely different influence mechanism in violet and green LD electron leakage. In fact, the electron current leakage is mainly related to its energy band structure. The impact of changing Al content of AlGaN EBL in violet and green LDs on band structure will be analyzed in the following content.

**Figure 3:**
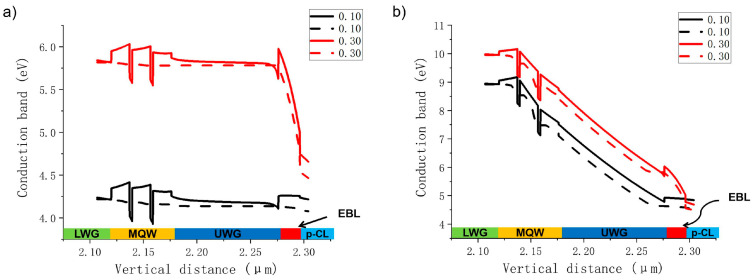
A comparison of the band structure (solid line) and Fermi level (dashed line) of the violet LD in the EBL when Al content is 0.10 and 0.30 (**a**) and the green LD in the EBL when the Al content is 0.10 and 0.30 (**b**).

To better explore the influence mechanism of Al content of the AlGaN EBL on electron leakage of violet and green LDs, the Al content is roughly divided into two ranges: the lower Al content (0.03 to 0.25) range and the higher Al content (0.25 to 0. 5) range. Contents of 0.10 in the lower Al range and 0.30 in the higher Al range are selected for comparison in the following analysis. The band maps of violet and green LDs under an injection current of 120 mA are calculated. The comparison diagram of the violet LD energy band is shown in Figure 3a, and the energy band comparison diagram of the green LD is shown in Figure 3b. Generally, a higher effective electron barrier height at the EBL can lead to a better suppression of electron leakage. The effective barrier height of an electron is defined as the difference between the conduction band and the local Fermi-level position. In the case of the violet LD, the effective barrier height of electrons at the EBL is 121 meV for the AlGaN EBL when the Al content is 0.10 and 221 meV when the Al content is 0.30. As Al content increases to 0.30, the height of effective electron blocking at the EBL increases by 82.6%, and the percentage of electron current leakage decreases by 97.8%. Increased height of the effective electron barrier can effectively prevent more electrons from overflowing into the P-type region. It is also observed from the figure that an increase in Al content can cause increased band bending at the EBL. This phenomenon appears to be due to the increased polarization effect caused by the increased Al content of the EBL. When the Al content continues to increase, the energy band at the EBL becomes more and more tilted, and the original rectangular energy band is seriously deformed into a sharp, triangular energy band structure. At this time, its influence is equivalent to a great reduction in the effective thickness of the EBL, and electrons can tunnel through the potential barrier easier to overflow the EBL. It effectively illustrates why the PELC does not decrease further increases when the Al content of the EBL is increased much higher for the violet LD.

In the case of the green LD, the effective barrier height of electrons at the EBL is 294 meV for the AlGaN EBL when the Al content is 0.10 and 228 meV when the Al content is 0.30. As the Al content increases to 0.30, the height of effective electron blocking at the EBL is reduced by 22.4%, and the percentage of electron current leakage is increased by 35.14 times. The aggravation of electron leakage is mainly due to the fact that the conduction band at the interface between the EBL and the InGaN UWG bends seriously downward below the Fermi level as a result of the polarization electric field, which leads to an accumulation of electrons at the interface. Some of the electrons eventually overflow from the EBL interface to the P-type region under a high-current condition. Therefore, the overall results show that for the violet LD, the electron leakage is the smallest when the Al content of the EBL is 0.25. For the green LD, in order to realize less electron leakage, the Al content of the EBL should be low.

#### 3.1.2. Effects of Al Content in the AlGaN EBL on Electrical and Optical Properties of LDs

**Figure 4:**
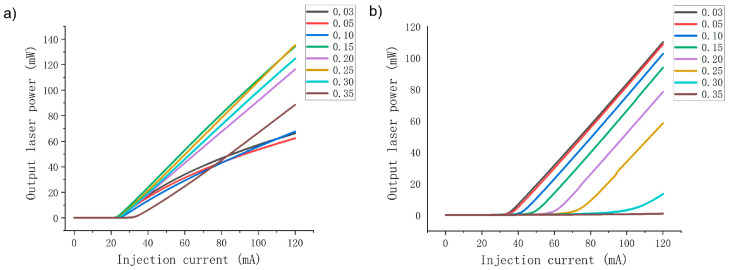
The output power of the violet LD changes with increasing current when the Al content of the EBL changes in the range of 0.03 to 0.35 (**a**). The output power of the green LD changes with increasing current when the Al content of the EBL changes in the range of 0.03 to 0.35 (**b**).

Moreover, for the purpose of better identifying the influence of Al content in the EBL on the output performance of the LDs, the changes in corresponding electrical and optical characteristics of the two LDs are studied when the AlGaN EBL Al content increases from 3% to 35%. The P–I curves of violet and green LDs are shown in Figure 4. It can be seen that the P–I curves of violet and green LDs show distinct differences when the Al content in the EBL increases. For the violet LD, when the EBL Al content increases from 0.03 to 0.25, the output power at an injection current of 120 mA is greatly enhanced from 66.2 mW to 135.4 mW. In this range, the slope efficiency is also significantly improved from 0.87 to 1.43, corresponding to an increase of 64.4%. However, as the Al content continues to increase to 0.35, the output power and slope efficiency of the violet light LD decrease significantly, and the threshold current increases considerably. The results show that the variation trend of the P–I curve of the violet LD is consistent with the variation trend of the electron leakage, as mentioned above. The reduction in electron leakage under low Al content improves the electrical performance of the violet LD, and the aggravation of electron leakage in under high Al content leads to a deterioration of the electrical performance.

However, for the green LD, as shown in Figure 4b, with increased EBL Al content, both its output power and slope efficiency decrease, especially in the case of high Al content. In theory, electrical performance should be improved due to the decrease in electron leakage in the high Al content range, but the calculation results show a serious decline in electrical performance. The reasons for this phenomenon will be discussed later. The variation trend of the green LD in the low Al range is in accordance with the variation trend of electron leakage, that is, intensification of electron leakage, which mainly deteriorates the electrical and optical properties of the green LD.

**Figure 5:**
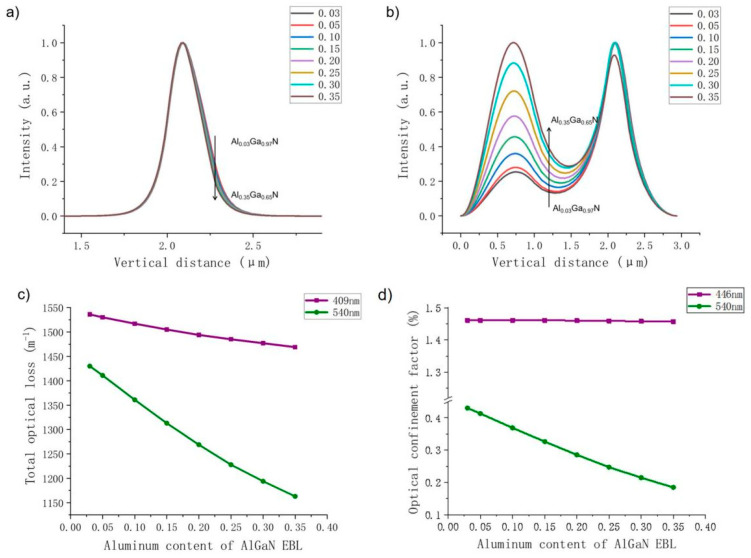
Violet LD optical field distribution when Al content in the EBL varies from 0.03 to 0.35 (**a**); green LD (**b**); comparison of the total optical loss of the violet (purple) and green (green) LDs (**c**); comparison of optical confinement factors of the violet and green LDs (**d**).

Next, we discuss the effects of different EBL Al contents of AlGaN on the optical confinement properties of violet and green LDs. Figure 5 depicts a comparison of optical field distribution, total optical loss and optical confinement factors when Al content changes for violet and green LDs. For the violet LD, with an increase in EBL Al content, the refractive index contrast between the EBL and UWG increases, and the center of the optical field moves away from the P-type region. Since the optical field distribution gradually moves away from the high-absorption layer, optical loss decreases when the Al content increases.

For the green LD, there are two peaks shown in the distribution of the optical field; the left one represents the optical field leaked into the GaN substrate, and the one on the right corresponds to the optical field distributed around the active region. It is known that optical field leakage is a serious issue for green LDs because as the wavelength increases, the refractive index contrast between LWG and n-CL decreases, resulting in a weakening of optical field confinement and an increase in light field leakage to the substrate. Figure 5 shows that the higher the Al content of the EBL, the more serious the light field leakage problem. Even when the Al fraction is 0.35, the center of the light field is shifted to the vicinity of the substrate. This is because as the refractive index of the EBL decreases due to the increase in Al content, the difference between the EBL and UWG refractive indices becomes larger, and the light field center moves farther away from the P-type region. Compared with the violet LD, because the center of the optical field of the green LD is farther away from the high-absorbing layers, the optical loss decreases more significantly. In contrast to the violet LD, the optical confinement factor of the green light LD decreases significantly, and the proportion of the optical field distribution in the active region is greatly reduced. This means that the optical confinement is greatly reduced, resulting in a rapid deterioration of the output power, slope efficiency and threshold current of the green LD under high Al content. At this time, the electron current density injected into the quantum wells decreases from 4627 A/cm^3^ under an Al content of 0.03 to 2278 A/cm^3^ under an Al content of 0.30. The reduction significantly affects the value of the PELC. This means that low PELC under high Al content does not contribute to better electrical performance but the opposite.

### 3.2. Blue and Cyan LDs

**Figure 6:**
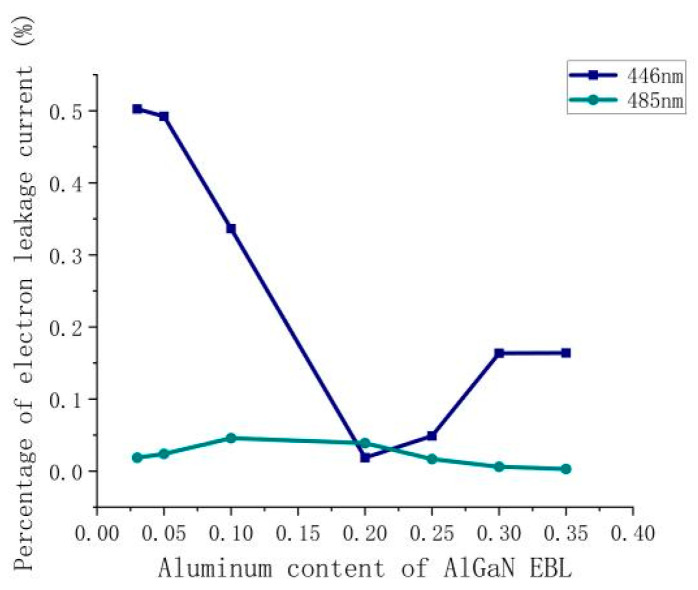
Relationship between the percentage of electron leakage current of blue and cyan LDs and the Al content of the AlGaN EBL.

**Figure 7:**
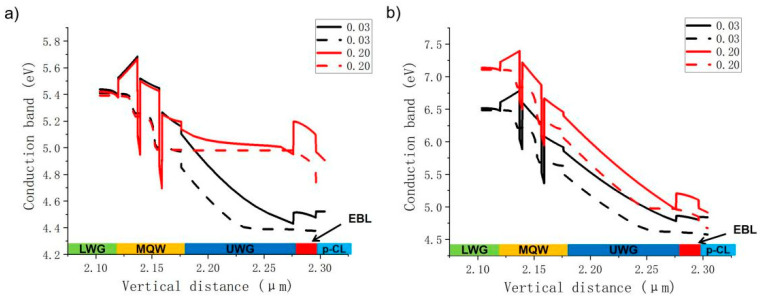
Band structure comparison of the blue LD in the EBL under Al contents of 0.03 and 0.20 (**a**); band structure comparison of the cyan LD in the EBL under Al contents of 0.03 and 0.20 (**b**).

In this section, in order to verify the influencing mechanism of the EBL Al content on LDs of different colors, the PELCs of a blue LD (laser wavelength = 446 nm) and a cyan LD (laser wavelength = 485 nm) are studied, as shown in Figure 6. Under low Al content (0.03–0.20), the electron leakage issue of the blue LD is dramatically ameliorated by increasing the Al content from 50.2% to 1.8%. The PELC of the cyan LD increases slightly with an increase in Al content from 1.8% to 3.9%. When the Al content is high (0.20~0.35), a further increase in Al content induces opposite variation trends in the two LDs. The PELC of the blue LD increases to 16.4%, but the PELC of the cyan LD decreases further to 0.3%. According to the numerical data, the variation trend of electron leakage percentage of blue and cyan LDs is smaller than that of violet and green LDs. Meanwhile, the band structures of blue and cyan LDs were also simulate under EBL Al contents of 0.03 and 0.20, respectively. The results are shown in Figure 7. For the blue LD, when the Al content of the AlGaN EBL is 0.03, the effective electron barrier height at the EBL is 126 meV; when the Al content is 0.20, the effective electron barrier height at the EBL increases by 81% to 228 meV, and the electron current leakage percentage decreases by 96.3%. When the Al content of the AlGaN EBL is 0.03, the height of the effective electron barrier at the EBL is 248 meV, and when the Al content is 0.20, the height of the effective electron barrier at the EBL decreases to 244 meV, and the PELC is doubled. The results show that the overall variation trend of the PELC of two LDs is the same as that of the violet and green LDs, as described in Section 3.1.

**Figure 8:**
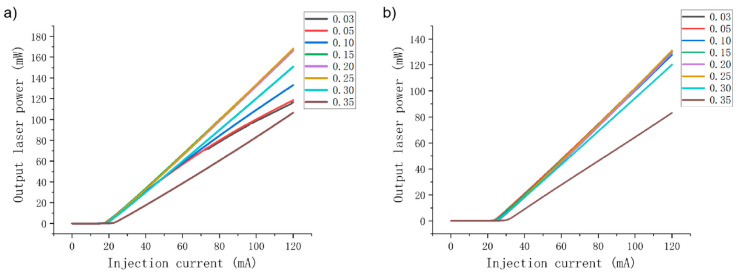
The output power of the blue LD versus the injection current when the Al content of the EBL changes in the range of 0.03 to 0.35 (**a**); the output power of the cyan LD versus injection current when the Al content of the EBL changes in the range of 0.03 to 0.35 (**b**).

**Figure 9:**
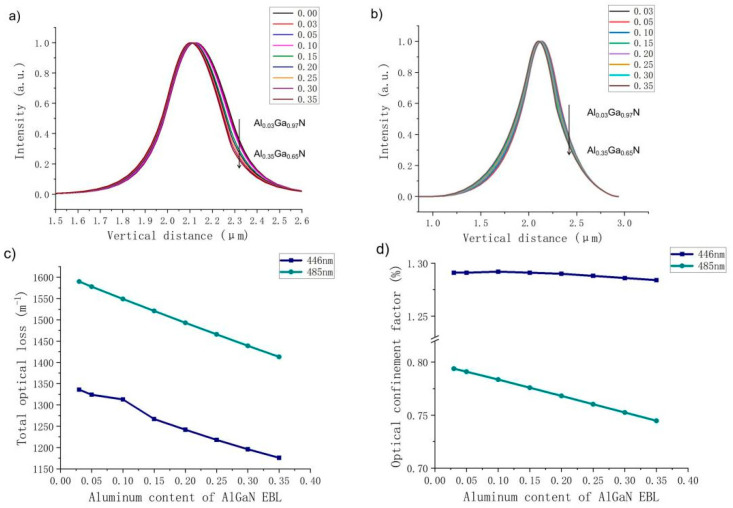
Optical field distribution of blue (**a**) and cyan (**b**) LDs when the Al content in the EBL varies from 0.03 to 0.35; comparison of the total optical loss of blue and cyan LDs (**c**); comparison of optical confinement factors of blue and cyan LDs (**d**).

Subsequently, the electrical and optical properties of the blue and cyan LDs are also calculated. Figure 8 illustrates their P–I curves as the EBL Al content increases. It can be seen from the figure that both the output power and slope efficiency of the blue LD increase when the Al content increases under lower Al contents. If the Al content is high, the output power and slope efficiency of blue LD decrease with increased Al content. The output power and slope efficiency of the cyan LD decrease as the Al content increases. On the other hand, according to the diagrams of optical field distribution, optical loss and optical confinement shown in Figure 9, it is clear that the optical properties of the blue and cyan LDs are closer to those of the violet and green LDs, respectively. As the Al content increases, the optical confinement of the blue LD is enhanced, and the optical field distribution becomes more concentrated, while the optical field of the cyan LD gradually shifts to the substrate. Therefore, their optical loss and optical confinement factor vary in correspondence with those of violet and green LDs, respectively. In fact, because the laser wavelength of the cyan LD is much closer to that of the green LD, the optical confinement factor of the green LD is significantly lower than that of the blue LD.

Through the above discussion, it can be found that since the laser wavelength of the blue LD is close to that of the violet LD, the influencing mechanism of the EBL Al content on electron leakage and the output performance of the blue LD is similar to that of the violet LD, and the cyan LD is similar to green LD due to the closeness of their wavelengths. Therefore, it can be concluded that the EBL Al content can significantly modulate electron leakage of LDs and change the output performance. However, the influence gradually decreases as the laser emission wavelength increases.

## 4. Conclusions

In summary, we used LASTIP to systematically investigate the influencing mechanism of Al content of the AlGaN EBL on GaN-based QW LD performance at different wavelengths. It was found that the modulation of the EBL Al content is more effective in improving short-wavelength LD performances. The shorter the wavelength, the greater the influence; therefore, a more appropriate Al content is required. For example, the optimal Al content was 0.25 for the violet LD (λ = 409 nm) and 0.15 for the blue LD (λ = 446 nm). Excessive Al content causes an increase in electron leakage. However, for LDs with longer wavelengths, the EBL Al content should be low. An analysis of the effect of the working mechanism of the EBL shows that LD performances are not only dependent on the change in effective barrier height but are also influenced by changes in the polarization effect and optical confinement induced by an additional EBL in the LD. Therefore, controlling the EBL Al content is beneficial in terms of reducing the leakage of carriers and improving the performance of LDs with different wavelengths.

## Data Availability

Data underlying the results presented in this paper may be obtained from the authors upon reasonable request.
